# The Target of Rapamycin and Mechanisms of Cell Growth

**DOI:** 10.3390/ijms19030880

**Published:** 2018-03-16

**Authors:** Andrew R. Tee

**Affiliations:** Division of Cancer and Genetics, Cardiff University, Heath Park, Cardiff CF14 4XN, UK; teea@cardiff.ac.uk

**Keywords:** mTOR, cell growth, rapamycin, protein translation, ribosomal biogenesis

## Abstract

Mammalian target of rapamycin (mTOR, now referred to as mechanistic target of rapamycin) is considered as the master regulator of cell growth. A definition of cell growth is a build-up of cellular mass through the biosynthesis of macromolecules. mTOR regulation of cell growth and cell size is complex, involving tight regulation of both anabolic and catabolic processes. Upon a growth signal input, mTOR enhances a range of anabolic processes that coordinate the biosynthesis of macromolecules to build cellular biomass, while restricting catabolic processes such as autophagy. mTOR is highly dependent on the supply of nutrients and energy to promote cell growth, where the network of signalling pathways that influence mTOR activity ensures that energy and nutrient homeostasis are retained within the cell as they grow. As well as maintaining cell size, mTOR is fundamental in the regulation of organismal growth. This review examines the complexities of how mTOR complex 1 (mTORC1) enhances the cell’s capacity to synthesis de novo proteins required for cell growth. It also describes the discovery of mTORC1, the complexities of cell growth signalling involving nutrients and energy supply, as well as the multifaceted regulation of mTORC1 to orchestrate ribosomal biogenesis and protein translation.

## 1. Introduction

### 1.1. History of Rapamycin and Drug Targets

The background history of the discovery of mechanistic target of rapamycin (mTOR) is atypical. More commonly, small molecule kinase inhibitors are developed after discovery of the protein kinase. However, in the case of mTOR, this protein kinase was discovered through the drug activity of a naturally occurring inhibitor called rapamycin. Rapamycin (also known as sirolimus and later marketed under the trade name Rapamune by Pfizer) is a macrocyclic lactone isolated from *Streptomyces hygroscopicus*, a bacterium extracted from a soil sample on Easter Island (known as ‘Rapa-Nui’) [1]. The biosynthesis of rapamycin is an energy intensive multi-step process involving many multifunctional enzymes (reviewed in [2]). The bacteria acquire a selective advantage by synthesising and secreting rapamycin into the soil. Rapamycin has antifungal properties, enabling the bacteria to more easily colonise the soil by repressing the growth of competing fungi [3]. Originally defined as an antifungal compound in the mid-70s, rapamycin was later found to be effective as an immunosuppressant with anti-proliferative properties in humans [4,5].

While the drug property of rapamycin to impair growth was beginning to be realised, the drug target was unknown until the early 1990s. The first breakthrough into the mechanism of drug activity was the finding that rapamycin interacted with FKBP12 (FK506-binding protein 12), an immunophilin in mammalian cells (reviewed in [6]). Following on from this, research in yeast led to a series of pivotal discoveries. By using FK506 columns, the yeast FKBP (FK506-binding protein) was purified and was identified as FK506-binding protein 1 (FRR1) [7]. Surprisingly, yeast genetics revealed that FRR1 was not involved in controlling cell growth. This was because genetic loss of *FRR1* did not arrest growth like rapamycin, implying that there had to be another drug target of rapamycin [4,7,8]. To identify this elusive drug target, genetic screens in yeast were carried out for rapamycin-resistant mutants [7,9]. These genetic screens did not only reveal *FRR1*, but also discovered two genes that were accordingly named as *target of rapamycin 1* (*TOR1*) and *TOR2*. Disruption of both *TOR1* and *TOR2* phenocopied rapamycin to cause cell growth arrest in yeast [10]. From these early studies, it was ascertained that rapamycin acted on TOR1 and TOR2 to repress cell growth. However, the other piece of the puzzle involving rapamycin-FRR1 (as a drug-immunophilin complex) was not yet recognised. For some drug-immunophilin complexes, drug association with an immunophilin results in adding an additional drug activity, which is observed when cyclosporine A interacts with FK506 [6]. Therefore, it was speculated that rapamycin might gain a new drug activity to arrest cell growth when associated with an FKBP. Substantiating this idea, the rapamycin-FRR1 complex in yeast was later found to bind directly to TOR1 and TOR2 to arrest cell growth [11,12,13]. In mammalian systems, it was also observed that a homologous protein to yeast TOR1 and TOR2 directly interacted with FKBP12-rapamycin [14,15], which was later called mTOR. By the mid-1990’s, the drug activity of rapamycin was better understood; rapamycin could bind to and inhibit TOR as a rapamycin-FKBP drug-immunophilin complex. In yeast, loss of *TOR1* and *TOR2* phenocopies nutrient starvation to cause inhibition of protein synthesis, glycogen accumulation and autophagy induction [16,17,18,19]). Since these early yeast studies, much has been discovered, including delineation of the mTOR signalling network and conservation of this pathway in higher eukaryotic cells.

### 1.2. mTOR Structure and Protein Complexes

mTOR is a member of the phosphatidylinositol 3-kinase-related kinases (PIKK) family, which includes DNA-dependent protein kinase (DNA-PK, also known as protein kinase, DNA-activated, catalytic polypeptide), ATM serine/threonine kinase (ATM) and ATR serine/threonine kinase (ATR). Instead of having lipid kinase activity, mTOR’s kinase catalytic domain functions to phosphorylate proteins on Ser/Thr residues [15,20]. Distinguishing features of mTOR include multiple HEAT (Huntington, EF3, A subunit of PP2A, TOR1) repeats within the N-terminus that permits multiple protein-protein interactions [21]. mTOR also possesses a central FAT (FRAP, ATM, TRAP) domain and a C-terminal FAT (FATC) domain that are also conserved in other PIKK family members [22]. Allosteric interaction of FKBP12-rapamycin with the FKBP-rapamycin binding (FRB) domain is inhibitory to the phosphotransferase activity of mTOR. The FRB domain is situated between the central FAT and the kinase domain [11,14].

While yeast possesses two *TOR* genes, there is only one *mTOR* gene in higher eukaryotes. Rather than gene duplication as an evolutionary route to generate two distinctive TOR protein kinases as observed in yeast, higher eukaryotes instead possess a single *mTOR* gene that becomes integral to two protein kinase complexes, called mTOR complex 1 (mTORC1) and mTORC2. Both mTORC1 and mTORC2 are high molecular weight protein complexes that contain an array of core binding and regulatory proteins that are associated with the mTOR catalytic subunit. mTORC1 was first identified by the association of mTOR with rapamycin-associated protein of TOR (Raptor) [23,24] and MTOR associated protein, LST8 homolog (mLST8) [25]. Raptor functions as a scaffold protein and it’s binding to mTOR is necessary for mTORC1 substrate specificity (reviewed in [26]). mTORC1 also interacts with regulatory proteins that inhibit mTORC1, proline-rich Akt substrate of 40 kDa (PRAS40) [27,28,29] and DEP domain containing mTOR-interacting protein (DEPTOR) [30]. Other regulatory mTORC1 interacting proteins include the telomere maintenance 2 (TELO2) and TELO2 interacting protein 1 (TTI1), which associates with mTORC1 as a TELO2/TTI1 complex and is necessary for mTORC1 assembly [31]. For mTORC2, mLST8 and DEPTOR also interact. Core binding subunits that are exclusive to mTORC2 include rapamycin-insensitive companion of mammalian target of rapamycin (Rictor), mSin1 (mammalian stress-activated protein kinase interacting protein 1) and Protor (protein observed with Rictor-1) [32,33]. Rictor is essential for substrate specificity of mTORC2 as well as its assembly and stability, while mSin1 functions as a negative regulator (reviewed in [34]). mTORC2 is a regulator of the cytoskeleton through its stimulation of F-actin stress fibers. mTORC2 directly phosphorylates and activates AKT to enhance cell growth via mTORC1. As mTORC1 is more centrally involved in the control of cell growth when compared to mTORC2, this review focuses mainly on mTORC1-regulated processes.

mTORC1 and mTORC2 can be distinguished by their differences in rapamycin sensitivity. Prior to the discovery of the two mTOR complexes, it was commonly presumed that rapamycin was effective at blocking the kinase activity of mTOR. However, the kinase activity of mTORC2 is insensitive to rapamycin, which was the principle reason why mTORC2-driven processes remained hidden in earlier studies that employed rapamycin. Furthermore, it is now appreciated that some mTORC1-mediated phosphorylation events are partially resistant to rapamycin [35,36]. As an example, mTORC1-mediated phosphorylation of a well-characterised mTORC1 substrate, eukaryotic initiation factor-4E-binding protein 1 (4E-BP1) at Thr37/Thr46 is heavily resistant to rapamycin treatment [37]. Another partially rapamycin resistant process that is regulated by mTORC1 is autophagy, where the level of resistance varies depending on cell-type [36]. It was hypothesised that cells with a more open (or relaxed) mTORC1 conformation would likely have higher sensitivity to rapamycin [36]. For mTORC2, short-term rapamycin treatment is not inhibitory. However, longer durations of treatment can indirectly inhibit mTORC2. This is because rapamycin-FKBP12 associates with newly synthesised mTOR prior to complex assembly, sequestering this “free pool” of mTOR from Rictor to prevent mTORC2 formation. Consequently, prolonged treatments of rapamycin (over days of treatment) can indirectly lead to reduced mTORC2 activity [38].

Rapamycin has been instrumental in the discovery of mTOR and has helped elucidate rapamycin sensitive cell processes that were later found to be driven by mTORC1. However, with the introduction of gene silencing and targeted genomic editing technologies, a much greater appreciation of mTORC1 and mTORC2 is beginning to emerge, where knockdown or knockout of either Raptor or Rictor is now more frequently used to ablate mTORC1 or mTORC2 signal transduction, respectively. Furthermore, the introduction of ATP-competitive inhibitors of mTOR adds an additional research tool, allowing researchers to more fully repress the kinase activity of both mTOR complexes. 

## 2. mTORC1 and Cell Growth Control

### 2.1. Upstream Signalling Pathway of mTORC1

Model systems such as *Drosophila* have been instrumental in understanding the role that mTOR has in cell growth control and organismal size. Genetic analysis of the *Drosophila* TOR homolog, *dTOR* (also now being referred to as mTOR), clearly revealed that inactivating *mTOR* mutations cause a delay in cell proliferation and reduce cell size [39]. While conversely, *mTOR* activation promoted cell and organ size [39]. Another key discovery made was the identification of the *Drosophila* genes *dTSC1* (Tuberous sclerosis complex 1) and *dTSC2* as regulators of cell size [40,41,42]. Genetic epistasis experiments uncovered that *dTSC1* and *dTSC2* functioned as negative regulators of cell size, positioning them downstream of the insulin receptor and AKT (AKT serine/threonine kinase) [40,41]. These genetic studies positioned *Drosophila ribosomal protein S6 kinase* (dS6K) downstream of *dTSC1* and *dTSC2*. In fruit flies, dS6K controls cell size, where *dS6K* inactivation mutations cause a small fly phenotype [43]. dS6K, as well the mammalian homologue, ribosomal protein S6 kinase 1 (S6K1) are well-characterised rapamycin-sensitive substrates of mTORC1 [44]. These fly studies inferred that dTSC1 and dTSC2 functioned upstream of mTORC1. Sequential studies confirmed that *dTSC1*/*dTSC2* were genetically positioned upstream from *mTOR*. Inactivation mutations of *mTOR* blocked the large cell phenotype in *dTsc2* mutant flies [45]. Furthermore, mutations reducing the activity of mTOR and dS6K rescued the lethality caused by *dTSC1* mutations [42]. Therefore, it was discovered that dTSC1 and dTSC2 functioned to repress growth signals upstream of mTORC1.

**Figure 1:**
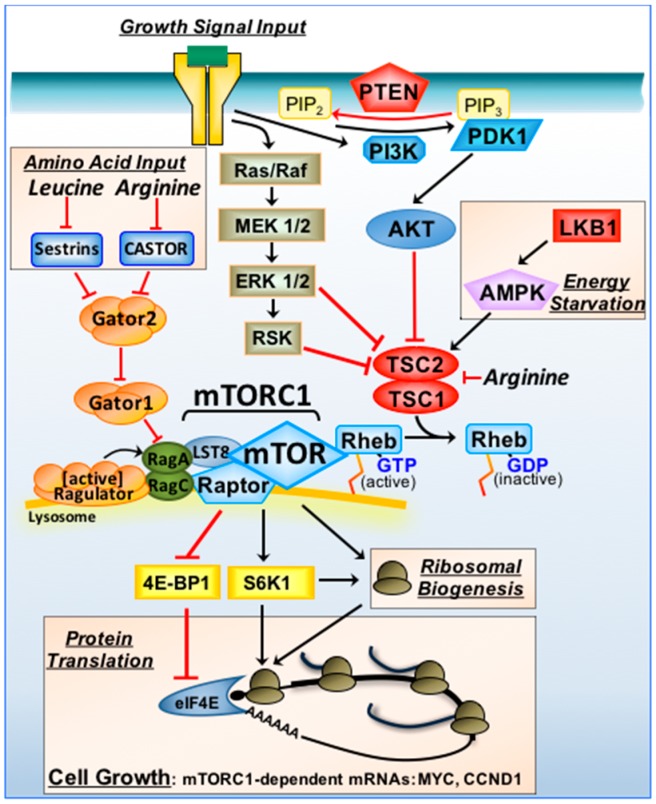
mTORC1 signal transduction and tumour suppressors. Growth signals via plasma membrane bound receptors activate the Ras/Raf/MAPK/ERK/RSK and PI3K/AKT signalling pathways. Tumour suppressors upstream of TSC1/TSC2 include PTEN and LKB1 (indicated in red). Through these pathways, TSC1/TSC2 is inactivated, converting Rheb to an active GTP-bound state to promote mTORC1 (when associated with the “Ragulator complex” on lysosomal membranes). When nutrients are sufficient, Rag GTPase heterodimers recruit mTORC1 to the “Ragulator complex”. Arginine inhibits both TSC1/TSC2 and CASTOR. Leucine activates GATOR2 indirectly via sestrins. Under energy deprivation, LKB1/AMPK activates TSC1/TSC2 to switch mTORC1 off. mTORC1 drives cell growth (in part) by increasing the efficiency of mRNA translation of mTORC1-sensitive mRNAs (that include MYC and CCND1). mTORC1 regulates protein synthesis via 4E-BP1/eIF4E and S6K1.

Tuberous sclerosis complex (TSC) is an autosomal dominant genetic condition caused by loss of function mutations in either *TSC1* or *TSC2*. *TSC1* and *TSC2* are tumour suppressors that function together, where loss of function mutations of either gene predisposes the growth of tumours in multiple organs in TSC patients, including the kidney, brain and skin, as well as neurocognitive problems and epilepsy [46]. Tumour growth in TSC patients is now known to be dependent on mTORC1. Consequently, primary care to treat TSC patients include the use of mTORC1 inhibitors, such as everolimus [46]. Basic research showing the involvement of TSC1/TSC2 in mTORC1 signal transduction followed by rapamycin pre-clinical studies in mouse models of TSC [47] were instrumental in the repositioning of mTORC1 inhibitors to treat TSC. Basic research that followed on from the genetic analysis in *Drosophila* described above revealed a conserved pathway in mammals. Within the phosphatidylinositide 3-kinase (PI3K)/AKT signalling pathway, AKT directly phosphorylates TSC2 on four or five residues, revealing that TSC1/TSC2 is directly regulated by AKT [48,49,50]. Over-expression of TSC1 and TSC2 also markedly inhibited S6K1, positioning TSC1/TSC2 upstream of S6K1. TSC1/TSC2 was then later shown to repress signal transduction through mTORC1 [51]. It was assumed that a small-G protein was likely regulated by TSC1/TSC2, as TSC2 (also known as tuberin) possessed a conserved C-terminal GTPase activating protein (GAP) domain that was commonly lost in TSC patients (via C-terminal truncating point-nonsense mutations) [52,53]. Furthermore, a cluster of pathogenic single point mutations within the conserved GAP domain of TSC2 was also reported [53]. In yeast, a likely small G-protein candidate was Ras homologue enriched in brain (Rheb), where genetic loss of Rheb in yeast phenocopied nutrient starvation [54]. Confirming the involvement of this small G protein, Rheb was found to be regulated by TSC1/TSC2 in mammalian cells [55,56,57]. When complexed with TSC1, TSC2 functions as a Rheb GAP, switching Rheb to an inactive GDP-bound form to turn mTORC1 off [57]. Rheb becomes activated, i.e., GTP-loaded, when the TSC1/TSC2 complex is repressed through an array of upstream kinases within mitogenic and hormone stimulated pathways (depicted in Figure 1). Paralleling the PI3K/AKT pathway, mitogen-activated protein kinases (MAPK) also converges on TSC1/TSC2 to positively regulate mTORC1 [58]. It was later found that activation of MAPK also inhibits TSC1/TSC2 through TSC2 phosphorylation by RSK [59] and ERK [60].

### 2.2. Regulation of Protein Translation and Cell Growth by mTORC1

mTORC1 relays nutrient, energy and growth signals to drive cell growth through promotion of anabolic processes such as protein synthesis. As well as enhancing the efficiency of protein translation, mTORC1 promotes protein translation through increasing the production of ribosomes. Furthermore, mTORC1 boosts the generation of nucleotide precursors that are essential for a growing cell. These nucleotide precursors are required for the generation of rRNA to build new ribosomes, mRNA to be transcribed into proteins necessary for growth and dNTP nucleotides for DNA replication during cell division.

mTORC1 regulates protein translation through an array of translation factors that include 4E-BP1 and S6K1. Both 4E-BP1 and S6K1 possess an mTORC1 signalling (TOS) motif, which associates with Raptor [61]. Consequently, the TOS motif is necessary for the recruitment of both 4E-BP1 and S6K1 to mTORC1 and the phosphorylation of these substrates [61]. 4E-BP1 acts as a negative regulator of cap-dependant protein translation. eukaryotic initiation factor (eIF) 4E associates with the m^7^GpppN cap moiety on the 5′-end of mRNAs and is inactivated through association with 4E-BP1 when in an unphosphorylated state, thus preventing the translation of mRNAs involved in cell growth [62]. mTORC1 phosphorylates 4E-BP1 on four Ser/Thr residues, causing dissociation of 4E-BP1 from eIF4E. As association of either 4E-BP1 or eIF4G to eIF4E are mutually exclusive, eIF4G then associates with eIF4E to promote translation initiation (reviewed in [21]). eIF4G acts as a scaffold to recruit other translation initiation factors, such as eIF4A, to form the eIF4F complex. Assembly of eIF4F is considered a rate-limiting step of translation initiation. As part of the eIF4F complex, eIF4A has RNA helicase activity that unwinds the secondary structure within the 5′-untranslated region (5′-UTR) of the mRNA to help the efficacy of ribosome scanning to the start codon. Via mTORC1, S6K1 further enhances the RNA helicase activity of eIF4A by phosphorylating eIF4B on Ser422 [63]. mRNAs involved in cell growth are heavily dependent on mTORC1. The length and degree of secondary structure within the 5’-UTR contributes to the dependency of these mRNAs to mTORC1 and the availability of eIF4F (reviewed in [64]). Examples of mRNAs involved in cell growth that are highly dependent on eIF4F include the MYC proto-oncogene, bHLH transcription factor (MYC) and cyclin D1 (CCND1) [65].

### 2.3. Ribosomal Biogenesis and mTORC1

The cells’ capacity to generate protein is limited by the number of ribosomes they have. Consequently, mTORC1 promotes ribosomal biogenesis to ensure rapid growth. Typically, 5′-terminal oligopyrimidine (5′-TOP) tracts are found in mRNA that encode for factors involved in ribosomal biogenesis and ribosomal proteins [66,67]. These 5′-TOP tracts function as translational *cis*-regulatory elements. 5′-TOP mRNAs are sensitive to rapamycin and are found to be dependent on S6K1 [68]. Over 75% of the proteins involved in ribosomal biogenesis are estimated to be controlled by S6K1 [68]. In a recent study, high resolution ribosomal profiling identified 144 mRNAs that were sensitive to mTORC1 inhibitors [69]. Only 68% of these mRNAs possessed putative 5′-TOP tracts, while 63% contained a newly discovered *cis*-regulatory pyrimidine-rich translational element (PRTE). Rather than being dependent on S6K1, PRTE mRNAs were found to be highly sensitive to 4E-BP1. Many of the mTORC1-sensitive target genes uncovered by Hsieh et al., were ribosomal proteins [69], which highlights the critical involvement of mTORC1 in the generation of new ribosomes. The regulatory mechanism of these translational *cis*-regulatory elements is currently unknown. Presumably, trans-acting factors bind to these elements and regulate the translation of the mRNA. Several potential trans-acting factors that bind to the 5′-TOP have been studied to date that include the La protein [70] and the La-related proteins (LARP) [71,72,73].

mTORC1 also promotes the generation of rRNA. rRNAs are the major component of the ribosome that comprise of 60% of the ribosomal mass. Eukaryotic ribosomes consist of four rRNAs called the 5S, 5.8S, 18S and 28S. RNA polymerase I (Pol I) transcribes a precursor 47S pre-rRNA that is sequentially processed in the nucleoli to form the mature 5.8S, 18S and 28S rRNA species. The 5S rRNA is generated by Pol III, which is also involved in the formation of transfer RNA (tRNA). To further enhance the production of rRNA, mTORC1 activates both Pol I and Pol III via several mechanisms. mTORC1 directly phosphorylates Transcription Initiation Factor I (TIF1A) to cause its translocation to the nucleolus and activation of Pol I [74]. mTORC1 further promotes Pol I via S6K1. S6K1 phosphorylates Upstream Binding Factor (UBF), causing UBF-TIF1B interaction and Pol I activation [75]. Through Pol I, mTORC1 and S6K1 enhance the production of 5.8S, 18S and 28S rRNA. mTORC1 also enhances the production of the 5S rRNA species by phosphorylating and inhibiting a negative regulator of Pol III, called MAF1 (MAF1 homolog, negative regulator of RNA polymerase III) [76]. In another study, mTORC1 was shown to directly bind to the promoter region of Pol I and Pol III, leading to their enhanced gene-expression [77]. Therefore, mTORC1-dependent regulation of ribosomal proteins and rRNA during ribosomal biogenesis is multifaceted.

To ensure that there is a sufficient supply of nucleotides for the generation of rRNA, mTORC1 promotes the production of nucleotide precursors. mTORC1 does this by redirecting glucose metabolites into the pentose phosphate pathway. The pentose phosphate pathway generates nicotinamide adenine dinucleotide phosphate, ribose-5-phosphate and erythrose-4-phosphate, precursors for fatty acids, nucleotides and aromatic amino acids, respectively. Generation of fatty acids, nucleotides and aromatic amino acids are essential for a growing cell, i.e., for the expansion of membranes and the de novo synthesis of mRNA, rRNA, DNA, and proteins. Carbamoyl-phosphate synthetase 2, aspartate transcarbamylase, and dihydroorotase (CAD) within the pentose phosphate pathway was found to be phosphorylated and activated by S6K1 [78]. CAD catalyses the first three enzymatic steps of a six-step pyrimidine biosynthesis pathway to enrich the pyrimidine nucleotide pool [78]. More recently, mTORC1 was found to enhance purine biosynthesis through ATF4-mediated gene-expression of methylenetetrahydrofolate dehydrogenase 2 (MTHFD2), a metabolic enzyme involved in the synthesis of purine nucleotides [79]. In this study, it was found that mTORC1 increased the protein translation of ATF4, the transcription factor that drives the gene-expression of MTHFD2.

## 3. Nutrient and Energy Homeostasis during Cell Growth

Cell growth is tightly controlled by both nutrient and energy supply. If the supply of either nutrients or energy is insufficient, feedback mechanisms ensure that mTORC1 is switched off. Such signalling mechanisms maintain energy and nutrient homeostasis during cell growth.

### 3.1. Nutrient Signalling and mTORC1

Early studies implicated that mTORC1 required the presence of branch-chained amino acids for its full activation (reviewed in [80]). However, it was unclear how nutrients were “sensed” by mTORC1. The first indication that mTORC1 sensed an intracellular amino acid pool were in experiments that utilised cycloheximide, a drug that blocks translation elongation [81]. The amino acid reserves of a cell can become quickly exhausted via protein translation. Therefore, inhibition of protein translation with cycloheximide can be used to help indirectly replenish the intracellular amino acid stores. It was found that increasing the pool of intracellular amino acids after cyclohexmide treatment was sufficient to activate mTORC1 in the absence of external amino acid supply or growth factor stimuli [81]. It is now appreciated that intracellular nutrient sensing occurs at the level of lysosomes. In summary, Rag GTPase heterodimers regulate the localisation of mTORC1 to lysosomes [82,83]. During amino acid sufficiency, active Rag heterodimers (consisting of either RagA-GTP or RagB-GTP bound to either RagC-GDP or RagD-GDP) will associate with Raptor. These active Rag heterodimers bound to Raptor then translocate mTORC1 to the “Ragulator complex” found on the membrane surface of lysosomes, which is necessary for mTORC1 activation [84]. Under conditions of nutrient withdrawal, mTORC1 is cytoplasmically localised and is inactive. The Rag proteins are regulated by the Ragulator complex that is comprised of five proteins referred to as the late endosomal/lysosomal adaptor, MAPK and mTOR activator 1-5 (LAMTOR1-5) [84]. The Ragulator complex acts as a guanine nucleotide exchange factor (GEF) towards RagA and RagB, switching them to an active GTP-bound state [85]. RagA and RagB are negatively regulated by RagGTPases and GTRs-1 (GATOR) and GATOR2, that are also lysosomally localised [86]. GATOR1 acts as a RagGAP, switching both RagA and RagB to an inactive GDP-bound state to prevent lysosomal localisation of mTORC1 (to turn off mTORC1 signalling). GATOR2 lies directly upstream of GATOR1 and functions as a negative regulator of GATOR1. GATOR2 is positively regulated by nutrients [86].

Leucine was proposed to be sensed by three sestrins (SESN1-3) that activate GATOR2, which then in turn inactivates GATOR1 to switch the Rag heterodimers to an active state and promote mTORC1 signalling [87,88]. However, there is some controversy regarding SESN1-3 as a direct leucine sensor (reviewed in [89]). This is due to the known function of SESN1-3 to activate AMPK during cell stress [90,91,92]. Activation of AMPK can indirectly turn off mTORC1 (via a signalling feedback mechanism involving TSC1/2 that is summarised in Section 3.2 below). Indeed, genetic evidence in SESN-deficient fly and mouse models infer that the overriding function of SESN is to activate AMPK and to inhibit mTORC1, which importantly can occur in the presence of physiological levels of leucine [90,91,92]. It is possible that leucine is sensed by Leucyl-tRNA synthetase at the lysosome instead, which has been shown to function as a GAP towards RagD [93].

Arginine is sensed by mTORC1 via several mechanisms. It was recently found that TSC1/2 associates with Rheb when arginine levels are low, which causes TSC1/2 to switch Rheb to an inactive GDP-bound state to inhibit mTORC1 [94]. As another mechanism of arginine sensing, cytosolic arginine sensor for mTORC1 (CASTOR) subunit 1 interacts with GATOR2 in the absence of arginine to prevent mTORC1 activation [95]. When arginine levels are in sufficient supply, arginine binds to CASTOR1 to displace GATOR2 from the inhibited CASTOR1-CATOR2 complex. Consequently, GATOR2 is then able to turn off GATOR1, leading to mTORC1 activation [95]. Further studies in animal models will be required to validate these nutrient sensing mechanisms.

Evidently, nutrient sensing by mTORC1 is complex and involves multiple inputs. In the context of growth control, leucine is well known to play a significant role in the determination of muscle mass involving mTORC1. Leucine accounts for about 20% of our dietary protein intake and branch-chained amino acids account for about a third of muscle protein [96]. Muscle is the primary reservoir of protein within the human body. Therefore, it is not surprising that muscle cells are subjected to particularly high levels of protein turnover rates that help maintain amino acid homeostasis within the whole body. Leucine is critical for driving muscle growth and does this, in part, through the activation of mTORC1. Resistance exercise with protein supplements (including leucine) can promote the build-up of muscle mass [97]. Through activation of mTORC1, leucine is a potent activator of protein synthesis that drives anabolic muscle growth.

Nutrient withdrawal is known to restrict organ size and implicates the mTORC1 pathway and nutrient sensing in organismal growth. Growth regulation of organs is complex, involving multiple pathways that coordinates cell number (via the control of proliferation and apoptosis) and cell size. It is appreciated that mTORC1 works alongside the Hippo pathway to coordinate organ growth (reviewed in [98]). The transcription factor yes-associated protein 1 (YAP1) is activated in the Hippo pathway to drive gene-expression of CCND1 and Myc, which are involved in proliferation and cell size control, respectively. It is interesting that the protein translation of CCND1 and Myc mRNAs are mTORC1-dependent (Figure 1, and [65]). As a consequence, the Hippo and mTORC1 pathways would appear to work jointly to enhance organ growth by enhancing the transcriptional and translational regulation of CCND1 and Myc.

### 3.2. Energy Signalling and mTORC1

The anabolic processes needed for cellular growth are dependent on energy as well as nutrients, so mTORC1 enhances mitochondrial biogenesis to generate more mitochondria [99], ensuring that the cell has enough capacity to generate energy as the cell grows. The ability of mTORC1 to switch between states of anabolic growth and catabolic fasting is dynamically regulated and is intrinsically coupled with the energy senor, AMP-dependant protein kinase (AMPK). A major catabolic process that AMPK regulates is autophagy (reviewed in [100]). In the disease setting, improper mTORC1 signalling leads to uncontrolled cell growth and loss of energy homeostasis. Energy stress, through activation of AMPK is also upstream of TSC1/TSC2, where AMPK-dependent phosphorylation of TSC2 on Thr1227 and Ser1345 activates the TSC1/TSC2 tumour suppressor, leading to mTORC1 inhibition [101]. AMPK also phosphorylates Raptor, which is also inhibitory to mTORC1 [102]. AMPK becomes active when ATP levels decline and AMP levels increase. Upon energy stress, AMPK is activated by the serine/threonine kinase LKB1/STK11 [103,104]. Inactivating mutations of LKB1 inhibits AMPK and can give rise to Peutz-Jeghers syndrome that predisposes patients to hamartomatous polyps and cancer in the gastrointestinal tract [105]. As cells require a plentiful supply of energy to grow efficiently, this negative signalling input from AMPK ensures that cell growth through mTORC1 is halted when energy supply is insufficient. To quickly restore energy balance within the cell, AMPK not only downregulates anabolic processes driven by mTORC1, but also enhances catabolic processes, such as autophagy.

## 4. Conclusions

Since the discovery of rapamycin, much has been learnt about its drug target, mTORC1, and the role it plays in cell growth control. mTORC1 is regulated at the level of lysosomes, where multiple signalling inputs from growth factor (and hormone) receptors as well as from nutrients and energy all converge onto mTORC1. Coordination of such signalling inputs ensures that the homeostatic balance of nutrients and energy is maintained as a cell grows. The capacity of a cell to manufacture de novo proteins is rate limiting in cell growth, which is why the regulation of protein synthesis by mTORC1 is multifaceted through ribosomal biogenesis, generation of nucleotide precursors, and the regulation of protein translation. mTORC1 signalling is clearly tightly integrated with metabolism, but there is still much to understand. The nuclear function of mTORC1 is also currently unknown. Underdeveloped areas of research involving mTORC1 includes chromosomal remodelling and the regulation of transcription factors linked to cell growth control. mTORC1 is also implicated in mRNA splicing that adds another layer of regulation [106]. Clearly, there is still much to be discovered regarding how mTORC1 ‘mechanistically’ controls cell growth.
